# Machine Learning Model for Predicting Risk of In-Hospital Mortality after Surgery in Congenital Heart Disease Patients

**DOI:** 10.31083/j.rcm2311376

**Published:** 2022-11-03

**Authors:** Xinwei Du, Hao Wang, Shunmin Wang, Yi He, Jinghao Zheng, Haibo Zhang, Zedong Hao, Yiwei Chen, Zhiwei Xu, Zhaohui Lu

**Affiliations:** ^1^Department of Cardiothoracic Surgery, Shanghai Children’s Medical Center, School of Medicine, Shanghai Jiaotong University, 200127 Shanghai, China; ^2^Information Center, Shanghai Children’s Medical Center, School of Medicine, Shanghai Jiaotong University, 200127 Shanghai, China; ^3^Shanghai Synyi Medical Technology Co., Ltd. 201203 Shanghai, China

**Keywords:** congenital heart disease, in-hospital mortality, machine learning, Extreme Gradient Boosting

## Abstract

**Background:**

A machine learning model was developed to estimate the in-hospital mortality risk after congenital heart disease (CHD) surgery in pediatric patient.

**Methods:**

Patients with CHD who underwent surgery were included in the study. A Extreme Gradient Boosting (XGBoost) model was constructed based onsurgical risk stratification and preoperative variables to predict the risk of in-hospital mortality. We compared the predictive value of the XGBoost model with Risk Adjustment in Congenital Heart Surgery-1 (RACHS-1) and Society of Thoracic Surgery-European Association for Cardiothoracic Surgery (STS-EACTS) categories.

**Results:**

A total of 24,685 patients underwent CHD surgery and 595 (2.4%) died in hospital. The area under curve (AUC) of the STS-EACTS and RACHS-1 risk stratification scores were 0.748 [95% Confidence Interval (CI): 0.707–0.789, *p <* 0.001] and 0.677 (95% CI: 0.627–0.728, *p <* 0.001), respectively. Our XGBoost model yielded the best AUC (0.887, 95% CI: 0.866–0.907, *p <* 0.001), and sensitivity and specificity were 0.785 and 0.824, respectively. The top 10 variables that contribute most to the predictive performance of the machine learning model were saturation of pulse oxygen categories, risk categories, age, preoperative mechanical ventilation, atrial shunt, pulmonary insufficiency, ventricular shunt, left atrial dimension, a history of cardiac surgery, numbers of defects.

**Conclusions:**

The XGBoost model was more accurate than RACHS-1 and STS-EACTS in predicting in-hospital mortality after CHD surgery in China.

## 1. Introduction

Congenital heart disease (CHD) is the most common congenital malformations. The prevalence of CHD at birth is about 75–90/10,000 for live births and total pregnancies, with CHD occurring in approximately 1% of live births and 10% of aborted fetuses [1, 2]. In addition, CHD is the leading cause of mortality in children with birth defects [3] and affects 0.7% children born in China [4]. The risk of mortality in Chinese children with CHD has been increasing [5].

Surgery has been the cornerstone in the treatment of patients with CHD [6]. Without interventions, patients with CHD will experience significant mortality. In developed countries, surgery has greatly improved the outcome of patients with CHD and significantly reduced the mortality rate [7]. However, approximately 20% children who undergo surgery for pediatric CHD are readmitted within 30 days, and 4.2% patients who undergo surgery for CHD die [8, 9]. Early mortality after cardiac surgery in the neonatal period is approximately 10% [1].

Risk of death in CHD patients is associated with complexity of surgical procedures [10]. Accurate prediction of in-hospital death is important to facilitate clinical decisions-making for the performance of certain procedures and improve patient’s outcome [11]. Several major risk stratification categories are currently available for the prediction of mortality and morbidity in children undergoing surgery for CHD—Risk Adjustment in Congenital Heart Surgery-1 (RACHS-1) [12], Aristotles Basic Complexity, and Aristotles Comprehensive Complexity [13], Society of Thoracic Surgery-European Association for Cardiothoracic Surgery (STS-EACTS) Congenital Heart Surgery (STAT) Mortality Categories [14]. These risk adjustment categories have been developed based on projections of risk or complexity and heavily rely on expert experience and consensus [15]. These traditional tools focus on surgical procedure categories and do not include sufficient individual patient risk factors. Therefore, it may have lower predictive accuracy for individual patients. The prognosis should be determined by combined analysis of multiple features. Thus, it is of great clinical significance to build a prediction model that includes multiple important clinical features.

Some studies have shown performance of machine learning-assisted tools were better than standard scoring systems [16, 17]. Machine learning has the advantage of flexibility and scalability compared to traditional biostatistical methods [18]. It is well suited for complex multidimensional data and may uncover interactions that hard to be identified and illustrated through classic statistical analysis [19]. Extreme Gradient Boosting (XGBoost) is a machine learning algorithm. It is an implementation of Gradient Boosting that was originally started as a research project by Tianqi Chen as part of the Distributed (Deep) Machine Learning Community (DMLC) group at the University of Washington [20]. Currently being the fastest and the best open source boosting tree toolkit, XGBoost has made many optimizations, such as significant improvements in model training speed and accuracy. Kilic evaluated the predictive performance of XGBoost model for risk of death after cardiac surgery, and found that the XGBoost model was superior in predictive performance compared to Society of Thoracic Surgeons Predicted Risk of Mortality (STS-PROM) score [21]. Zeng *et al*. [22] showed that a XGBoost model has better prediction performance for predicting postoperative complications than other traditional risk adjustment models after paediatric cardiac surgery. However, research on the application of machine learning model for the prediction of mortality risk in children with CHD is lacking, especially in China.

The aim of the study was to establish and validate a XGBoost model for predicting the in-hospital mortality risk in pediatric CHD surgery, and to compare the predictive value of the XGBoost model with the RACHS-1 and STS-EACTS categories.

## 2. Methods

### 2.1 Study Design and Population

Patients aged 0–18 years who were diagnosed with CHD and underwent CHD surgery at Shanghai Children’s Medical Center, School of Medicine, Shanghai Jiaotong University between January 1, 2006 and December 31, 2017 were included. For patients with multiple surgical records within a month, only the information of the last surgical record was extracted, and the previous surgical records were regarded as “operation history”. The exclusion criteria included general thoracic surgery (not involving cardiac surgery), patients with incomplete or missing in-hospital survival records, and surgical procedures that were performed in less than 3 patients. Our study was approved by the Ethical Committee of Shanghai Children’s Medical Center, School of Medicine, Shanghai Jiaotong University. As our study only involved a retrospective review of previous clinical data, the requirement for informed consent was waived.

### 2.2 Data Source and Extraction

The database was constructed by merging information from multiple data sources, including the laboratory information management system, hospital information system, intensive care unit database, clinical data repository, and the surgical record database of the cardiac surgery department in Shanghai Children’s Medical Center. We built a feature engineering pipeline to load and transform clinical data during and before CHD surgery for each individual. The collected data were divided into five categories as follows: (i) demographic data, such as sex, body mass index, and age; (ii) preoperative clinical factors, including diagnosis, numbers of defects, pulse oxygen saturation, a history of cardiac surgery (any prior cardiac surgeries), numbers of defects, non-cardiac malformations, and other risk factors; (iii) complexity of the CHD surgery according to RACHS-1 and STS-EACTS morbidity categories; (iv) cardiac Doppler ultrasound data; and (v) preoperative laboratory test results, including routine blood test findings, liver function test results, and coagulation index. Variables with more than 30% missing values were excluded.

### 2.3 In-Hospital Mortality and Estimation of Mortality Rates

The study endpoint was in-hospital mortality, defined as death due to any cause during hospitalization after surgery. The cause of death was defined as the disease, situation, or occurrence that causes a series of events, ultimately result in death [23]. And the cause of death in this study included cardiac, peri-operative, vascular and non-cardiovascular causes. Cardiac deaths included sudden death, documented ventricular arrhythmias, heart failure, infective endocarditis and myocardial infarction [23, 24]. Vascular death included haemorrhage, stroke, rupture of aneurysm, pulmonary embolism, and dissection [23, 24]. Non-cardiovascular death included malignancy, pneumonia, sepsis (excluding endocarditis), other infections, peritonitis, hip fracture, renal failure, suicide, and unknown [23, 24].

Mortality risk stratification was performed by classifying the procedures into clusters based on estimated mortality, following the statistical method proposed by a previous study [14]. First, we used a Bayesian random effect model to calculate the posterior probability distribution of the mortality rates of all procedures. Second, a homogeneity criterion was used to evaluate a partition scheme, which measured the within-category homogeneity of the mortality rates. The optimal partition solution to maximize the homogeneity criterion can be achieved using a dynamic programming algorithm. Finally, we successively performed the abovementioned calculations for 2–20 categories to determine the number of categories. The optimal category number was determined using of the Bayesian information criterion, a trade-off between homogeneity and partition complexity. All procedures were finally categorized into five relatively homogeneous categories. According to the pseudo-code algorithm description (see Appendix of the previous study) [14] we implemented handcrafted codes of the stratification computation pipeline using Python language (version 3.7.6, Python Software Foundation, Wilmington, DE, USA).

### 2.4 Construction of an In-Hospital Death Predictive Model Using a Machine Learning Algorithm

**Fig. 1.**
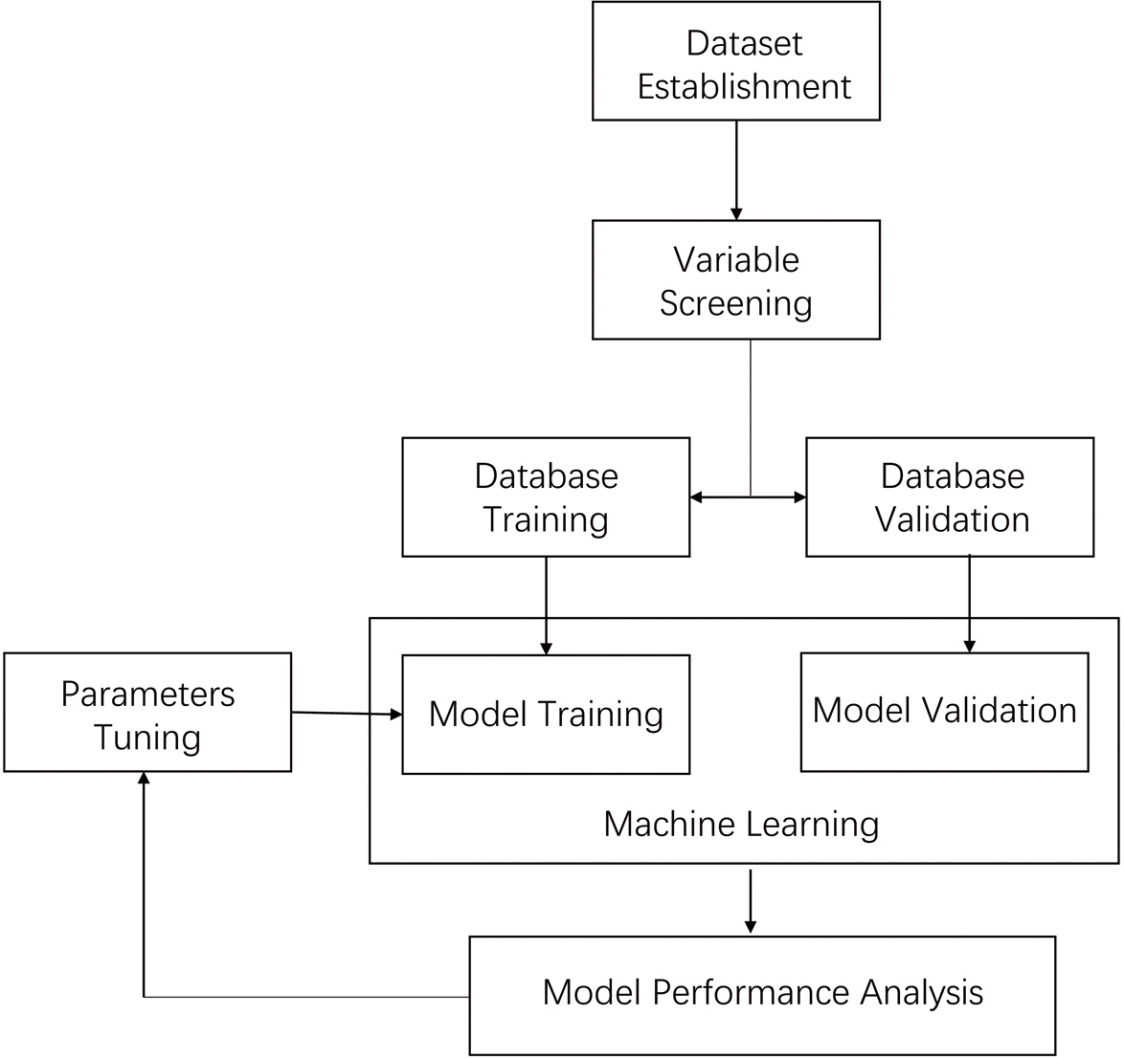
**Steps in the XGBoost model development**.

We used the XGBoost algorithm to build a in-hospital mortality predictive model for children with CHD. Dataset was devided into training set and testing set according to the 7.5:2.5 ratio. The training dataset was used for feature selection and model training, while the testing dataset was used for validation after model training. The importance of each feature was assessed using the recursive feature elimination (RFE) algorithm, and all features were sorted based on their level of importance. The RFE algorithm was used to recursively remove features and build a model on the remaining features. Among all possible combination of features, the model with the highest AUC was determined and the features included are eventually selected to build the XGBoost model. Furthermore, Grid Search was used to adjust the hyperparameters of model to reduce overfitting and improve the model accuracy. The stability of the model is tested by Bootstrap algorithm with random resampling of the samples, and 95% confidence interval (95% CI) was exported. Finally, we assessed the predictive power of the model using the area under the receiver operating characteristic curves (AUC), sensitivity, and specificity. Fig. 1 presents the whole process described above. The XGBoost was developed in Python language (version 3.7.6) with main packages scikit-learn (version 0.23.1) and XGBoost (version 1.1.1).

### 2.5 Statistical Analysis

Continuous variables are described as the median (range); all were non-normally distributed. Categorical variables are described using frequency (%). To assess the distributive balance between the training and validation sets, comparisons between groups were performed using the Mann–Whitney U test, Fisher’s exact test, and the chi-square test, as appropriate. The Area under receiver operating characteristic (ROC) curve (AUC) with 95% confidence interval (CI) was calculated to evaluate the predictive power. In addition, the optimal threshold was chosen by maximizing the Youden Index. The sensitivity and specificity of the predictive model were obtained based on the threshold. All statistical tests were two sided, and *p*-value of <0.05 was considered statistically significant. All analyses were performed using SAS software (SAS Institute Inc., Cary, NC, USA), version 4.2.0.

## 3. Results

### 3.1 Characteristics and Stratification of Surgical Procedure

A total of 24,685 patients underwent surgery for CHD were included (Table 1). The mean age of the patients was 316 (1–6568) days, and 14,215 (57.59%) patients were male. A total of 591 (2.4%) in-hospital deaths occurred. Other patient characteristics are summarized in Table 1.

**Table 1.** **Demographic and preoperative characteristics of patients**.

Parameter		Training set n = 18552				Testing set n = 6133				Total n = 24685
		Death n = 442	Non-death n = 18110	Total n = 18552	*p*-value	Death n = 149	Non-death n = 5984	Total n = 6133	*p*-value	
Age (days)*	Median (range)	168 (2,6459)	323 (1,6568)	317 (1,6568)	<0.001	153 (1,4716)	319 (2,6410)	313 (1,6410)	<0.001	316 (1,6568)
	<365 days	329 (74.4%)	9681 (53.5%)	10010 (54.0%)	<0.001	112 (75.2%)	3205 (53.6%)	3317 (54.2%)	<0.001	13327 (54.0%)
	365 days to 1095 days	51 (11.5%)	4600 (25.4%)	4651 (25.1%)		19 (12.8%)	1493 (25.0%)	1512 (24.7%)		6163 (25.0%)
	>1095 days	62 (14.0%)	3812 (21.1%)	3874 (20.9%)		18 (12.1%)	1276 (21.45)	1294 (21.1%)		5168 (21.0%)
Sex*	Male	278 (62.9%)	10342 (57.11%)	10620 (57.24%)	0.015	81 (54.36%)	3514 (58.72%)	3595 (58.62%)	0.286	14215 (57.59%)
	Female	164 (37.1%)	7768 (42.89%)	7932 (42.76%)		68 (45.64%)	2470 (41.28%)	2538 (41.38%)		10470 (42.41%)
BMI*	<P5 percentile	82 (20.55%)	3077 (19.26%)	3159 (19.29%)	0.494	34 (25.19%)	1066 (20.14%)	1100 (20.26%)	0.122	4259 (19.53%)
	P5–P95 percentile	252 (63.16%)	10539 (65.97%)	10791 (65.9%)		75 (55.56%)	3395 (64.13%)	3470 (63.92%)		14261 (65.41%)
	>P95 percentile	65 (16.29%)	2360 (14.77%)	2425 (14.81%)		26 (19.26%)	833 (15.73%)	859 (15.82%)		3284 (15.06%)
A history of cardiac surgery	0	311 (70.4%)	16323 (90.1%)	16634 (89.7%)	<0.001	103 (69.1%)	5364 (89.6%)	5467 (89.1%)	<0.001	22101 (89.5%)
	1	122 (27.6%)	1663 (9.2%)	1785 (9.6%)		44 (29.5%)	583 (9.7%)	627 (10.2%)		2412 (9.8%)
	>2	9 (2.0%)	124 (0.7%)	133 (0.7%)		2 (1.3%)	37 (0.6%)	39 (0.6%)		172 (0.7%)
Preoperative ICU admission	0	429 (97.1%)	17530 (96.8%)	17959 (96.8%)	0.758	141 (94.6%)	5755 (96.2%)	5896 (96.1%)	0.335	23855 (96.6%)
	1	13 (2.9%)	580 (3.2%)	593 (3.2%)		8 (5.4%)	229 (3.8%)	237 (3.9%)		830 (3.4%)
ABO blood type	A	125 (29.9%)	5534 (30.87%)	5659 (30.85%)	0.919	44 (31.21%)	1840 (30.97%)	1884 (30.98%)	0.988	7543 (30.88%)
	B	119 (28.47%)	4942 (27.57%)	5061 (27.59%)		38 (26.95%)	1654 (27.84%)	1692 (27.82%)		6753 (27.65%)
	O	95 (22.73%)	4210 (23.49%)	4305 (23.47%)		34 (24.11%)	1366 (22.99%)	1400 (23.02%)		5705 (23.36%)
	AB	79 (18.9%)	3238 (18.07%)	3317 (18.08%)		25 (17.73%)	1081 (18.2%)	1106 (18.18%)		4423 (18.11%)
RH blood type	-	3 (0.72%)	77 (0.43%)	80 (0.44%)	0.431	0 (0%)	26 (0.44%)	26 (0.43%)	>0.999	106 (0.43%)
	+	413 (99.28%)	17866 (99.57%)	18279 (99.56%)		140 (100%)	5904 (99.56%)	6044 (99.57%)		24323 (99.57%)
Premature	No	437 (98.87%)	17960 (99.17%)	18397 (99.16%)	0.423	145 (97.32%)	5927 (99.05%)	6072 (99.01%)	0.06	24469 (99.12%)
	Yes	5 (1.13%)	150 (0.83%)	155 (0.84%)		4 (2.68%)	57 (0.95%)	61 (0.99%)		216 (0.88%)
Non-cardiac malformation	No	440 (99.55%)	18088 (99.88%)	18528 (99.87%)	0.111	149 (100%)	5982 (99.97%)	6131 (99.97%)	>0.999	24659 (99.89%)
	Yes	2 (0.45%)	22 (0.12%)	24 (0.13%)		0 (0%)	2 (0.03%)	2 (0.03%)		26 (0.11%)
Chromosome abnormality or syndrome	No	438 (99.1%)	17920 (99.0%)	18358 (99.0%)	>0.999	146 (98.0%)	5920 (98.9%)	6066 (98.9%)	0.222	24424 (98.9%)
	Yes	4 (0.9%)	190 (1.0%)	194 (1.0%)		3 (2.0%)	64 (1.1%)	67 (1.1%)		261 (1.1%)
Allergy*	No	436 (98.64%)	17522 (96.75%)	17958 (96.8%)	0.026	145 (97.32%)	5783 (96.64%)	5928 (96.66%)	0.819	23886 (96.76%)
	Yes	6 (1.36%)	588 (3.25%)	594 (3.2%)		4 (2.68%)	201 (3.36%)	205 (3.34%)		799 (3.24%)
Special treatment before surgery*	No	400 (90.5%)	17800 (98.29%)	18200 (98.1%)	<0.001	136 (91.28%)	5876 (98.2%)	6012 (98.03%)	<0.001	24212 (98.08%)
	Yes	42 (9.5%)	310 (1.71%)	352 (1.9%)		13 (8.72%)	108 (1.8%)	121 (1.97%)		473 (1.92%)
Preoperative HCT*	Low	19 (5.4%)	873 (5.84%)	892 (5.83%)	<0.001	9 (6.87%)	327 (6.56%)	336 (6.57%)	0.069	1228 (6.02%)
	Normal	285 (80.97%)	13580 (90.92%)	13865 (90.69%)		112 (85.5%)	4470 (89.7%)	4582 (89.6%)		18447 (90.41%)
	High	48 (13.64%)	484 (3.24%)	532 (3.48%)		10 (7.63%)	186 (3.73%)	196 (3.83%)		728 (3.57%)
STS-EACTS categories*	1	111 (25.3%)	10359 (57.4%)	10470 (56.6%)	<0.001	27 (18.2%)	3405 (57.0%)	3432 (56.1%)	<0.001	13902 (56.5%)
	2	120 (27.4%)	5074 (28.1%)	5194 (28.1%)		44 (29.7%)	1708 (28.6%)	1752 (28.6%)		6946 (28.2%)
	3	117 (26.7%)	1407 (7.8%)	1524 (8.2%)		40 (27.0%)	443 (7.4%)	483 (7.9%)		2007 (8.2%)
	4	80 (18.3%)	1198 (6.6%)	1278 (6.9%)		33 (22.3%)	409 (6.8%)	442 (7.2%)		1720 (7.0%)
	5	10 (2.3%)	20 (0.1%)	30 (0.2%)		4 (2.7%)	6 (0.1%)	10 (0.2%)		40 (0.2%)
RACHS-1 categories	1	65 (14.7%)	3148 (17.4%)	3213 (17.3%)	<0.001	19 (12.8%)	966 (16.1)	985 (16.1%)	<0.001	4198 (17.0%)
	2	161 (36.4%)	11341 (62.6%)	11502 (62.0%)		45 (30.2%)	3830 (64.0%)	3875 (63.2%)		15377 (62.3%)
	3	152 (34.4%)	3124 (17.3%)	3276 (17.7%)		69 (46.3%)	1032 (17.2%)	1101 (18.0%)		4377 (17.7%)
	4	50 (11.3%)	446 (2.5%)	496 (2.7%)		12 (8.1)	135 (2.3%)	147 (2.4%)		643 (2.6%)
	5	8 (1.8%)	46 (0.3%)	54 (0.3%)		3 (2.0%)	20 (0.3%)	23 (0.4%)		77 (0.3%)
	6	6 (1.4%)	5 (0.0%)	11 (0.1%)		1 (0.7%)	1 (0.0%)	2 (0.0%)		13 (0.1%)
Risk stratification	1	28 (6.33%)	10605 (58.56%)	10633 (57.31%)	<0.001	14 (9.4%)	3459 (57.8%)	3473 (56.63%)	<0.001	14106 (57.14%)
	2	87 (19.68%)	4399 (24.29%)	4486 (24.18%)		33 (22.15%)	1477 (24.68%)	1510 (24.62%)		5996 (24.29%)
	3	145 (32.81%)	2228 (12.3%)	2373 (12.79%)		45 (30.2%)	717 (11.98%)	762 (12.42%)		3135 (12.7%)
	4	82 (18.55%)	529 (2.92%)	611 (3.29%)		27 (18.12%)	199 (3.33%)	226 (3.68%)		837 (3.39%)
	5	100 (22.62%)	349 (1.93%)	449 (2.42%)		30 (20.13%)	132 (2.21%)	162 (2.64%)		611 (2.48%)
Pulse oxygen saturation*	≤90%	235 (72.76%)	3368 (24.77%)	3603 (25.89%)	<0.001	80 (68.38%)	1179 (26.09%)	1259 (27.16%)	0.001	4862 (26.2%)
	>90%	88 (27.24%)	10228 (75.23%)	10316 (74.11%)		37 (31.62%)	3340 (73.91%)	3377 (72.84%)		13693 (73.8%)
Diameter of aortic sinus*	<Critical value	72 (25.9%)	3225 (20.83%)	3297 (20.92%)	0.008	22 (26.19%)	1018 (19.9%)	1040 (20%)	0.353	4337 (20.69%)
	Normal	72 (25.9%)	5299 (34.23%)	5371 (34.08%)		27 (32.14%)	1732 (33.85%)	1759 (33.83%)		7130 (34.02%)
	>Critical value	134 (48.2%)	6958 (44.94%)	7092 (45%)		35 (41.67%)	2366 (46.25%)	2401 (46.17%)		9493 (45.29%)
AAO*	Median (range)	1.1 (0.2, 4.11)	1.2 (0.4, 14.1)	1.2 (0.2, 14.1)	<0.001	1.15 (0.5, 6.69)	1.2 (0.4, 6.62)	1.2 (0.4, 6.69)	0.635	1.2 (0.2, 14.1)
DAO*	Median (range)	1.2 (0.6, 4.28)	1.3 (0.38, 11.1)	1.3 (0.38, 11.1)	0.005	1.2 (0.6, 3.45)	1.27 (0.5, 5.03)	1.23 (0.5, 5.03)	0.058	1.28 (0.38, 11.1)
MPA (mm)*	Median (range)	1.72 (0.34, 6.19)	1.6 (0.2, 20)	1.6 (0.2, 20)	0.022	1.7 (0.6, 17)	1.6 (0.3, 41.6)	1.6 (0.3, 41.6)	0.588	1.6 (0.2, 41.6)
MV (m/s)*	Median (range)	1 (0.3, 3.3)	1.2 (0.36, 3.81)	1.2 (0.3, 3.81)	<0.001	1 (0.4, 2.68)	1.2 (0.4, 12)	1.2 (0.4, 12)	<0.001	1.2 (0.3, 12)
TV2 (m/s)*	Median (range)	0.8 (0.4, 2.47)	0.8 (0.2, 33)	0.8 (0.2, 33)	0.025	0.8 (0.4, 1.7)	0.8 (0.3, 10.7)	0.8 (0.3, 10.7)	0.487	0.8 (0.2, 33)
Left atrial dimension*	<Critical value	79 (27.82%)	1907 (12.24%)	1986 (12.52%)	<0.001	25 (27.78%)	621 (12.06%)	646 (12.33%)	<0.001	2632 (12.47%)
	Normal	79 (27.82%)	3330 (21.37%)	3409 (21.49%)		26 (28.89%)	1146 (22.25%)	1172 (22.37%)		4581 (21.71%)
	>Critical value	126 (44.37%)	10343 (66.39%)	10469 (65.99%)		39 (43.33%)	3383 (65.69%)	3422 (65.31%)		13891 (65.82%)
LVDD*	<Critical value	209 (56.64%)	5535 (33.42%)	5744 (33.92%)	<0.001	65 (50.39%)	1791 (32.81%)	1856 (33.21%)	<0.001	7600 (33.75%)
	Normal	81 (21.95%)	3337 (20.15%)	3418 (20.19%)		28 (21.71%)	1095 (20.06%)	1123 (20.1%)		4541 (20.16%)
	>Critical value	79 (21.41%)	7691 (46.43%)	7770 (45.89%)		36 (27.91%)	2573 (47.13%)	2609 (46.69%)		10379 (46.09%)
LVEF*	<Critical value	176 (48.22%)	8881 (53.7%)	9057 (53.58%)	0.022	67 (52.34%)	3050 (55.93%)	3117 (55.85%)	0.009	12174 (54.15%)
	Normal	108 (29.59%)	4846 (29.3%)	4954 (29.31%)		27 (21.09%)	1495 (27.42%)	1522 (27.27%)		6476 (28.8%)
	>Critical value	81 (22.19%)	2811 (17%)	2892 (17.11%)		34 (26.56%)	908 (16.65%)	942 (16.88%)		3834 (17.05%)
LVFS*	<Critical value	191 (52.33%)	8697 (52.59%)	8888 (52.58%)	0.096	68 (53.13%)	2993 (54.89%)	3061 (54.85%)	0.01	11949 (53.14%)
	Normal	102 (27.95%)	5209 (31.5%)	5311 (31.42%)		28 (21.88%)	1600 (29.34%)	1628 (29.17%)		6939 (30.86%)
	>Critical value	72 (19.73%)	2632 (15.91%)	2704 (16%)		32 (25%)	860 (15.77%)	892 (15.98%)		3596 (15.99%)
LVPWD	<Critical value	121 (33.06%)	4734 (28.61%)	4855 (28.7%)	0.146	36 (28.13%)	1590 (29.15%)	1626 (29.12%)	0.605	6481 (28.81%)
	Normal	101 (27.6%)	4635 (28.01%)	4736 (28%)		39 (30.47%)	1448 (26.54%)	1487 (26.63%)		6223 (27.66%)
	>Critical value	144 (39.34%)	7179 (43.38%)	7323 (43.3%)		53 (41.41%)	2417 (44.31%)	2470 (44.24%)		9793 (43.53%)
Large artery shunt*	None	220 (58.51%)	14125 (85.46%)	14345 (84.86%)	<0.001	72 (54.96%)	4669 (85.8%)	4741 (85.07%)	<0.001	19086 (84.91%)
	Left to right	108 (28.72%)	1728 (10.45%)	1836 (10.86%)		37 (28.24%)	564 (10.36%)	601 (10.78%)		2437 (10.84%)
	Right to left	12 (3.19%)	53 (0.32%)	65 (0.38%)		2 (1.53%)	21 (0.39%)	23 (0.41%)		88 (0.39%)
	Two-way	36 (9.57%)	623 (3.77%)	659 (3.9%)		20 (15.27%)	188 (3.45%)	208 (3.73%)		867 (3.86%)
Atrial shunt*	None	77 (19.74%)	6169 (36.95%)	6246 (36.55%)	<0.001	29 (21.64%)	2018 (36.6%)	2047 (36.25%)	<0.001	8293 (36.48%)
	Left to right	114 (29.23%)	7450 (44.62%)	7564 (44.27%)		42 (31.34%)	2408 (43.68%)	2450 (43.39%)		10014 (44.05%)
	Right to left	48 (12.31%)	617 (3.7%)	665 (3.89%)		10 (7.46%)	235 (4.26%)	245 (4.34%)		910 (4%)
	Two-way	151 (38.72%)	2461 (14.74%)	2612 (15.29%)		53 (39.55%)	852 (15.45%)	905 (16.03%)		3517 (15.47%)
Ventricular shunt*	None	106 (27.39%)	4582 (27.45%)	4688 (27.45%)	<0.001	34 (25.95%)	1472 (26.73%)	1506 (26.71%)	<0.001	6194 (27.27%)
	Left to right	17 (4.39%)	6000 (35.95%)	6017 (35.23%)		4 (3.05%)	1996 (36.24%)	2000 (35.47%)		8017 (35.29%)
	Right to left	5 (1.29%)	33 (0.2%)	38 (0.22%)		0 (0%)	9 (0.16%)	9 (0.16%)		47 (0.21%)
	Two-way	259 (66.93%)	6077 (36.41%)	6336 (37.1%)		93 (70.99%)	2030 (36.86%)	2123 (37.66%)		8459 (37.24%)
AI*	Negative	307 (75.8%)	13794 (81.81%)	14101 (81.66%)	0.002	98 (71.01%)	4517 (81.2%)	4615 (80.95%)	0.003	18716 (81.49%)
	Slight	66 (16.3%)	2110 (12.51%)	2176 (12.6%)		28 (20.29%)	698 (12.55%)	726 (12.73%)		2902 (12.63%)
	Mild	28 (6.91%)	831 (4.93%)	859 (4.97%)		11 (7.97%)	311 (5.59%)	322 (5.65%)		1181 (5.14%)
	Mild to moderate	2 (0.49%)	66 (0.39%)	68 (0.39%)		1 (0.72%)	16 (0.29%)	17 (0.3%)		85 (0.37%)
	Moderate	2 (0.49%)	31 (0.18%)	33 (0.19%)		0 (0%)	16 (0.29%)	16 (0.28%)		49 (0.21%)
	Moderate to severe	0 (0%)	14 (0.08%)	14 (0.08%)		0 (0%)	2 (0.04%)	2 (0.04%)		16 (0.07%)
	Severe	0 (0%)	16 (0.09%)	16 (0.09%)		0 (0%)	3 (0.05%)	3 (0.05%)		19 (0.08%)
MR*	Negative	129 (36.13%)	4475 (27.41%)	4604 (27.6%)	0.001	32 (26.23%)	1492 (27.71%)	1524 (27.68%)	0.297	6128 (27.62%)
	Slight	151 (42.3%)	7495 (45.91%)	7646 (45.83%)		53 (43.44%)	2456 (45.62%)	2509 (45.57%)		10155 (45.77%)
	Mild	52 (14.57%)	3168 (19.41%)	3220 (19.3%)		22 (18.03%)	1031 (19.15%)	1053 (19.12%)		4273 (19.26%)
	Mild to moderate	14 (3.92%)	651 (3.99%)	665 (3.99%)		3 (2.46%)	233 (4.33%)	236 (4.29%)		901 (4.06%)
	Moderate	7 (1.96%)	389 (2.38%)	396 (2.37%)		5 (4.1%)	123 (2.28%)	128 (2.32%)		524 (2.36%)
	Moderate to severe	1 (0.28%)	82 (0.5%)	83 (0.5%)		4 (3.28%)	27 (0.5%)	31 (0.56%)		114 (0.51%)
	Severe	3 (0.84%)	65 (0.4%)	68 (0.41%)		3 (2.46%)	22 (0.41%)	25 (0.45%)		93 (0.42%)
PI*	Negative	95 (32.09%)	2423 (15.11%)	2518 (15.42%)	<0.001	27 (25%)	811 (15.38%)	838 (15.57%)	0.668	3356 (15.45%)
	Slight	123 (41.55%)	9015 (56.21%)	9138 (55.95%)		46 (42.59%)	2993 (56.75%)	3039 (56.47%)		12177 (56.08%)
	Mild	67 (22.64%)	4343 (27.08%)	4410 (27%)		29 (26.85%)	1374 (26.05%)	1403 (26.07%)		5813 (26.77%)
	Mild to moderate	3 (1.01%)	88 (0.55%)	91 (0.56%)		3 (2.78%)	31 (0.59%)	34 (0.63%)		125 (0.58%)
	Moderate	8 (2.7%)	156 (0.97%)	164 (1%)		2 (1.85%)	60 (1.14%)	62 (1.15%)		226 (1.04%)
	Moderate to severe	0 (0%)	4 (0.02%)	4 (0.02%)		1 (0.93%)	3 (0.06%)	4 (0.07%)		8 (0.04%)
	Severe	0 (0%)	8 (0.05%)	8 (0.05%)		0 (0%)	2 (0.04%)	2 (0.04%)		10 (0.05%)
TR*	Negative	10 (2.83%)	235 (1.45%)	245 (1.47%)	0.033	5 (4.2%)	71 (1.33%)	76 (1.39%)	0.153	321 (1.45%)
	Slight	165 (46.74%)	8606 (52.92%)	8771 (52.79%)		55 (46.22%)	2845 (53.29%)	2900 (53.13%)		11671 (52.88%)
	Mild	128 (36.26%)	5930 (36.47%)	6058 (36.46%)		35 (29.41%)	1922 (36%)	1957 (35.86%)		8015 (36.31%)
	Mild to moderate	25 (7.08%)	832 (5.12%)	857 (5.16%)		16 (13.45%)	290 (5.43%)	306 (5.61%)		1163 (5.27%)
	Moderate	15 (4.25%)	468 (2.88%)	483 (2.91%)		7 (5.88%)	144 (2.7%)	151 (2.77%)		634 (2.87%)
	Moderate to severe	3 (0.85%)	87 (0.54%)	90 (0.54%)		1 (0.84%)	28 (0.52%)	29 (0.53%)		119 (0.54%)
	Severe	7 (1.98%)	103 (0.63%)	110 (0.66%)		0 (0%)	39 (0.73%)	39 (0.71%)		149 (0.68%)
White blood cell count*	Normal	258 (83.23%)	13271 (93.91%)	13529 (93.68%)	<0.001	101 (84.87%)	4390 (93.98%)	4491 (93.76%)	<0.001	18020 (93.7%)
	Abnormal	52 (16.77%)	861 (6.09%)	913 (6.32%)		18 (15.13%)	281 (6.02%)	299 (6.24%)		1212 (6.3%)
Lymphocyte count	Normal	93 (30%)	4199 (29.71%)	4292 (29.72%)	0.913	35 (29.41%)	1395 (29.86%)	1430 (29.85%)	0.916	5722 (29.75%)
	Abnormal	217 (70%)	9933 (70.29%)	10150 (70.28%)		84 (70.59%)	3277 (70.14%)	3361 (70.15%)		13511 (70.25%)
Monocyte count*	Normal	147 (47.42%)	9971 (70.56%)	10118 (70.06%)	<0.001	59 (49.58%)	3240 (69.35%)	3299 (68.86%)	<0.001	13417 (69.76%)
	Abnormal	163 (52.58%)	4161 (29.44%)	4324 (29.94%)		60 (50.42%)	1432 (30.65%)	1492 (31.14%)		5816 (30.24%)
Neutrophil count*	Normal	182 (58.71%)	10609 (75.07%)	10791 (74.72%)	<0.001	79 (66.39%)	3498 (74.87%)	3577 (74.66%)	0.036	14368 (74.7%)
	Abnormal	128 (41.29%)	3523 (24.93%)	3651 (25.28%)		40 (33.61%)	1174 (25.13%)	1214 (25.34%)		4865 (25.3%)
Eosinophil count*	Normal	148 (49.33%)	8182 (59.86%)	8330 (59.63%)	<0.001	56 (48.7%)	2749 (60.93%)	2805 (60.62%)	0.008	11135 (59.88%)
	Abnormal	152 (50.67%)	5487 (40.14%)	5639 (40.37%)		59 (51.3%)	1763 (39.07%)	1822 (39.38%)		7461 (40.12%)
Basophil cell count*	Normal	259 (86.33%)	13083 (95.71%)	13342 (95.51%)	<0.001	101 (87.83%)	4314 (95.61%)	4415 (95.42%)	<0.001	17757 (95.49%)
	Abnormal	41 (13.67%)	586 (4.29%)	627 (4.49%)		14 (12.17%)	198 (4.39%)	212 (4.58%)		839 (4.51%)
Lymphocyte ratio*	Normal	92 (29.68%)	3539 (25.04%)	3631 (25.14%)	0.063	41 (34.45%)	1188 (25.43%)	1229 (25.65%)	0.026	4860 (25.27%)
	Abnormal	218 (70.32%)	10593 (74.96%)	10811 (74.86%)		78 (65.55%)	3484 (74.57%)	3562 (74.35%)		14373 (74.73%)
Red blood cell count*	Normal	197 (63.55%)	11561 (81.81%)	11758 (81.42%)	<0.001	81 (68.07%)	3825 (81.87%)	3906 (81.53%)	<0.001	15664 (81.44%)
	Abnormal	113 (36.45%)	2571 (18.19%)	2684 (18.58%)		38 (31.93%)	847 (18.13%)	885 (18.47%)		3569 (18.56%)
Hemoglobin*	Normal	197 (63.55%)	9645 (68.25%)	9842 (68.15%)	0.079	82 (68.91%)	3246 (69.48%)	3328 (69.46%)	0.894	13170 (68.48%)
	Abnormal	113 (36.45%)	4487 (31.75%)	4600 (31.85%)		37 (31.09%)	1426 (30.52%)	1463 (30.54%)		6063 (31.52%)
Hematocrit	Normal	225 (71.88%)	9974 (70.54%)	10199 (70.57%)	0.606	91 (75.83%)	3317 (70.92%)	3408 (71.04%)	0.241	13607 (70.69%)
	Abnormal	88 (28.12%)	4165 (29.46%)	4253 (29.43%)		29 (24.17%)	1360 (29.08%)	1389 (28.96%)		5642 (29.31%)
Mean corpuscular volume*	Normal	230 (74.19%)	9646 (68.26%)	9876 (68.38%)	0.026	83 (69.75%)	3183 (68.13%)	3266 (68.17%)	0.708	13142 (68.33%)
	Abnormal	80 (25.81%)	4486 (31.74%)	4566 (31.62%)		36 (30.25%)	1489 (31.87%)	1525 (31.83%)		6091 (31.67%)
MHC/MCH (solid mass)*	Normal	192 (61.94%)	6948 (49.17%)	7140 (49.44%)	<0.001	72 (60.5%)	2299 (49.21%)	2371 (49.49%)	0.015	9511 (49.45%)
	Abnormal	118 (38.06%)	7184 (50.83%)	7302 (50.56%)		47 (39.5%)	2373 (50.79%)	2420 (50.51%)		9722 (50.55%)
MHC/MCHC (mass concentration)*	Normal	211 (68.06%)	10816 (76.54%)	11027 (76.35%)	0.001	77 (64.71%)	3589 (76.82%)	3666 (76.52%)	0.002	14693 (76.39%)
	Abnormal	99 (31.94%)	3316 (23.46%)	3415 (23.65%)		42 (35.29%)	1083 (23.18%)	1125 (23.48%)		4540 (23.61%)
RDW-SD*	Normal	39 (12.58%)	6021 (42.61%)	6060 (41.96%)	<0.001	21 (17.65%)	2028 (43.41%)	2049 (42.77%)	<0.001	8109 (42.16%)
	Abnormal	271 (87.42%)	8111 (57.39%)	8382 (58.04%)		98 (82.35%)	2644 (56.59%)	2742 (57.23%)		11124 (57.84%)
Platelet count*	Normal	235 (75.81%)	12706 (89.91%)	12941 (89.61%)	<0.001	100 (84.03%)	4220 (90.33%)	4320 (90.17%)	0.023	17261 (89.75%)
	Abnormal	75 (24.19%)	1426 (10.09%)	1501 (10.39%)		19 (15.97%)	452 (9.67%)	471 (9.83%)		1972 (10.25%)
Mean platelet volume*	Normal	211 (68.06%)	11008 (77.9%)	11219 (77.69%)	<0.001	81 (68.07%)	3625 (77.61%)	3706 (77.37%)	0.014	14925 (77.61%)
	Abnormal	99 (31.94%)	3123 (22.1%)	3222 (22.31%)		38 (31.93%)	1046 (22.39%)	1084 (22.63%)		4306 (22.39%)
APTT*	Normal	148 (48.21%)	9173 (66.12%)	7211 (83.06%)	0.019	60 (50.42%)	3065 (66.85%)	3125 (66.43%)	<0.001	12446 (65.9%)
	Abnormal	159 (51.79%)	4701 (33.88%)	1471 (16.94%)		59 (49.58%)	1520 (33.15%)	1579 (33.57%)		6439 (34.1%)
Prothrombin time*	Normal	138 (44.81%)	9721 (70.06%)	9321 (65.73%)	<0.001	47 (39.17%)	3236 (70.49%)	3283 (69.69%)	<0.001	13142 (69.55%)
	Abnormal	170 (55.19%)	4155 (29.94%)	4860 (34.27%)		73 (60.83%)	1355 (29.51%)	1428 (30.31%)		5753 (30.45%)
Total protein*	Normal	84 (27.54%)	6555 (47.44%)	6639 (47.02%)	<0.001	34 (28.81%)	2187 (47.85%)	2221 (47.37%)	<0.001	8860 (47.1%)
	Abnormal	221 (72.46%)	7261 (52.56%)	7482 (52.98%)		84 (71.19%)	2384 (52.15%)	2468 (52.63%)		9950 (52.9%)
Serum albumin*	Normal	205 (66.99%)	12128 (87.67%)	12333 (87.22%)	<0.001	81 (68.07%)	3984 (87.12%)	4065 (86.64%)	<0.001	16398 (87.08%)
	Abnormal	101 (33.01%)	1706 (12.33%)	1807 (12.78%)		38 (31.93%)	589 (12.88%)	627 (13.36%)		2434 (12.92%)
Serum globulin*	Normal	188 (61.64%)	10800 (78.2%)	10988 (77.84%)	<0.001	76 (64.41%)	3601 (78.8%)	3677 (78.43%)	<0.001	14665 (77.99%)
	Abnormal	117 (38.36%)	3011 (21.8%)	3128 (22.16%)		42 (35.59%)	969 (21.2%)	1011 (21.57%)		4139 (22.01%)
Albumin/globulin ratio*	Normal	205 (67.21%)	11065 (80.12%)	11270 (79.84%)	<0.001	76 (64.41%)	3648 (79.82%)	3724 (79.44%)	<0.001	14994 (79.74%)
	Abnormal	100 (32.79%)	2746 (19.88%)	2846 (20.16%)		42 (35.59%)	922 (20.18%)	964 (20.56%)		3810 (20.26%)
ALT*	Normal	234 (76.47%)	12334 (89.09%)	12568 (88.82%)	<0.001	94 (78.99%)	4055 (88.61%)	4149 (88.37%)	0.001	16717 (88.71%)
	Abnormal	72 (23.53%)	1510 (10.91%)	1582 (11.18%)		25 (21.01%)	521 (11.39%)	546 (11.63%)		2128 (11.29%)
Aspartate aminotransferase*	Normal	130 (42.62%)	7806 (56.39%)	7936 (56.09%)	<0.001	53 (44.54%)	2516 (54.98%)	2569 (54.72%)	0.024	10505 (55.75%)
	Abnormal	175 (57.38%)	6038 (43.61%)	6213 (43.91%)		66 (55.46%)	2060 (45.02%)	2126 (45.28%)		8339 (44.25%)
ALP*	Normal	292 (95.74%)	13601 (98.44%)	13893 (98.39%)	0.001	115 (97.46%)	4497 (98.38%)	4612 (98.36%)	0.444	18505 (98.38%)
	Abnormal	13 (4.26%)	215 (1.56%)	228 (1.61%)		3 (2.54%)	74 (1.62%)	77 (1.64%)		305 (1.62%)
L-γ-glutamyltransferase	Normal	202 (66.23%)	9082 (65.74%)	9284 (65.75%)	0.857	79 (66.95%)	2924 (63.97%)	3003 (64.04%)	0.505	12287 (65.32%)
	Abnormal	103 (33.77%)	4734 (34.26%)	4837 (34.25%)		39 (33.05%)	1647 (36.03%)	1686 (35.96%)		6523 (34.68%)
Total bilirubin*	Normal	140 (48.78%)	11207 (81.73%)	11347 (81.05%)	<0.001	64 (58.72%)	3648 (80.44%)	3712 (79.93%)	<0.001	15059 (80.77%)
	Abnormal	147 (51.22%)	2506 (18.27%)	2653 (18.95%)		45 (41.28%)	887 (19.56%)	932 (20.07%)		3585 (19.23%)
Direct bilirubin*	Normal	191 (67.73%)	11953 (90.28%)	12144 (89.81%)	<0.001	82 (77.36%)	3946 (90.32%)	4028 (90.01%)	<0.001	16172 (89.86%)
	Abnormal	91 (32.27%)	1287 (9.72%)	1378 (10.19%)		24 (22.64%)	423 (9.68%)	447 (9.99%)		1825 (10.14%)
Creatinine*	Normal	283 (92.48%)	13682 (98.89%)	13965 (98.76%)	<0.001	109 (91.6%)	4527 (98.97%)	4636 (98.79%)	<0.001	18601 (98.76%)
	Abnormal	23 (7.52%)	153 (1.11%)	176 (1.24%)		10 (8.4%)	47 (1.03%)	57 (1.21%)		233 (1.24%)
Uric acid*	Normal	150 (49.02%)	8159 (58.99%)	8309 (58.77%)	<0.001	52 (43.7%)	2656 (58.07%)	2708 (57.7%)	0.002	11017 (58.5%)
	Abnormal	156 (50.98%)	5673 (41.01%)	5829 (41.23%)		67 (56.3%)	1918 (41.93%)	1985 (42.3%)		7814 (41.5%)
Eosinophils/100 leukocytes*	Normal	194 (64.45%)	10744 (78.6%)	10938 (78.29%)	<0.001	67 (58.26%)	3572 (79.15%)	3639 (78.63%)	<0.001	14577 (78.38%)
	Abnormal	107 (35.55%)	2926 (21.4%)	3033 (21.71%)		48 (41.74%)	941 (20.85%)	989 (21.37%)		4022 (21.62%)
Monocytes/100 leukocytes*	Normal	274 (88.39%)	13121 (92.85%)	13395 (92.75%)	0.003	104 (87.39%)	4317 (92.4%)	4421 (92.28%)	0.043	17816 (92.63%)
	Abnormal	36 (11.61%)	1011 (7.15%)	1047 (7.25%)		15 (12.61%)	355 (7.6%)	370 (7.72%)		1417 (7.37%)
Neutrophils/100 leukocytes	Normal	72 (23.23%)	3404 (24.09%)	3476 (24.07%)	0.726	23 (19.33%)	1150 (24.61%)	1173 (24.48%)	0.185	4649 (24.17%)
	Abnormal	238 (76.77%)	10728 (75.91%)	10966 (75.93%)		96 (80.67%)	3522 (75.39%)	3618 (75.52%)		14584 (75.83%)
Basophils/100 leukocytes*	Normal	239 (79.4%)	12199 (89.26%)	12438 (89.05%)	<0.001	94 (81.74%)	4015 (88.98%)	4109 (88.8%)	0.015	16547 (88.99%)
	Abnormal	62 (20.6%)	1468 (10.74%)	1530 (10.95%)		21 (18.26%)	497 (11.02%)	518 (11.2%)		2048 (11.01%)
TFIC*	Normal	249 (80.84%)	12561 (90.65%)	12810 (90.44%)	<0.001	99 (82.5%)	4145 (90.4%)	4244 (90.2%)	0.004	17054 (90.38%)
	Abnormal	59 (19.16%)	1295 (9.35%)	1354 (9.56%)		21 (17.5%)	440 (9.6%)	461 (9.8%)		1815 (9.62%)
Main defect*										
TGA	0	405 (91.6%)	17645 (97.4%)	18050 (97.3%)	<0.001	138 (92.6%)	5825 (97.3%)	5963 (97.2%)	0.003	24013 (97.3%)
	1	37 (8.4%)	465 (2.6%)	502 (2.7%)		11 (7.4%)	159 (2.7%)	170 (2.8%)		672 (2.7%)
APVC	0	419 (94.8%)	17618 (97.3%)	18037 (97.2%)	0.002	146 (98.0%)	5833 (97.5%)	5979 (97.5%)	1	24016 (97.3%)
	1	23 (5.2%)	492 (2.7%)	515 (2.8%)		3 (2.0%)	151 (2.5%)	154 (2.5%)		669 (2.7%)
COA	0	431 (97.5%)	17521 (96.7%)	17952 (96.8%)	0.37	146 (98.0%)	5793 (96.8%)	5939 (96.8%)	0.632	23891 (96.8%)
	1	11 (2.5%)	589 (3.3%)	600 (3.2%)		3 (2.0%)	191 (3.2%)	194 (3.2%)		794 (3.2%)
IAA	0	433 (98.0%)	18023 (99.5%)	18456 (99.5%)	<0.001	145 (97.3%)	5951 (99.4%)	6096 (99.4%)	0.012	24552 (99.5%)
	1	9 (2.0%)	87 (0.5%)	96 (0.5%)		4 (2.7%)	33 (0.6%)	37 (0.6%)		133 (0.5%)
AVC	0	433 (98.0%)	17585 (97.1%)	18018 (97.1%)	0.284	143 (96.0%)	5820 (97.3%)	5963 (97.2%)	0.309	23981 (97.1%)
	1	9 (2.0%)	525 (2.9%)	534 (2.9%)		6 (4.0%)	164 (2.7%)	170 (2.8%)		704 (2.9%)
Single ventricle	0	421 (95.2%)	17844 (98.5%)	18265 (98.5%)	<0.001	144 (96.6%)	5893 (98.5)	6037 (98.4%)	0.084	24302 (98.4%)
	1	21 (4.8%)	266 (1.5%)	287 (1.5%)		5 (3.4%)	91 (1.5%)	96 (1.6%)		383 (1.6%)
DORV	0	415 (93.9%)	17608 (97.2%)	18023 (97.1%)	<0.001	134 (89.9%)	5821 (97.3%)	5955 (97.1%)	<0.001	23978 (97.1)
	1	27 (6.1%)	502 (2.8%)	529 (2.9%)		15 (10.1%)	163 (2.7%)	178 (2.9%)		707 (2.9%)
ASD	0	326 (73.8%)	11528 (63.7%)	11854 (63.9%)	<0.001	105 (70.5%)	3808 (63.6%)	3913 (63.8%)	0.086	15767 (63.9%)
	1	116 (26.2%)	6582 (36.3%)	6698 (36.1%)		44 (29.5%)	2176 (36.4%)	2220 (36.2%)		8918 (36.1%)
VSD	0	325 (73.5%)	10741 (59.3%)	11066 (59.6%)	<0.001	106 (71.1%)	3555 (59.4%)	3661 (59.7%)	0.004	14727 (59.7%)
	1	117 (26.5%)	7369 (40.7%)	7486 (40.4%)		43 (28.9%)	2429 (40.6%)	2472 (40.3%)		9958 (40.3%)
PFO	0	420 (95.0%)	16294 (90.0%)	16714 (90.1%)	<0.001	142 (95.3%)	5376 (89.8%)	5518 (90.0%)	0.028	22232 (90.1%)
	1	22 (5.0%)	1816 (10.0%)	1838 (9.9%)		7 (4.7%)	608 (10.2%)	615 (10.0%)		2453 (9.9%)
TOF	0	400 (90.5%)	16731 (92.4%)	17131 (92.3%)	0.14	142 (95.3%)	5508 (92.0%)	5650 (92.1%)	0.145	22781 (92.3%)
	1	42 (9.5%)	1379 (7.6%)	1421 (7.7%)		7 (4.7%)	476 (8.0%)	483 (7.9%)		1904 (7.7%)
PDA	0	355 (80.3%)	15892 (87.8%)	16247 (87.6%)	<0.001	118 (79.2%)	5272 (88.1%)	5390 (87.9%)	0.001	21637 (87.7%)
	1	87 (19.7%)	2218 (12.2%)	2305 (12.4%)		31 (20.8%)	712 (11.9%)	743 (12.1%)		3048 (12.3%)
Pulmonary hypertension	0	391 (88.5%)	15070 (83.2%)	15461 (83.3%)	0.003	121 (81.2%)	5008 (83.7%)	5129 (83.6%)	0.419	20590 (83.4%)
	1	51 (11.5%)	3040 (16.8%)	3091 (16.7%)		28 (18.8%)	976 (16.3%)	1004 (16.4%)		4095 (16.6%)
Numbers of defects*	0	232 (52.5%)	6418 (35.4%)	6650 (35.8%)	<0.001	74 (49.7%)	2161 (36.1%)	2235 (36.4%)	<0.001	8885 (36.0%)
	1	33 (7.5%)	4405 (24.3%)	4438 (23.9%)		18 (12.1%)	1380 (23.1%)	1398 (22.8%)		5836 (23.6%)
	2	58 (13.1%)	3918 (21.6%)	3976 (21.4%)		14 (9.4%)	1350 (22.6%)	1364 (22.2%)		5340 (21.6%)
	3	48 (10.9%)	1700 (9.4%)	1748 (9.4%)		17 (11.4%)	554 (9.3%)	571 (9.3%)		2319 (9.4%)
	4	44 (10.0%)	1059 (5.8%)	1103 (5.9%)		15 (10.1%)	332 (5.5%)	347 (5.7%)		1450 (5.9%)
	5	19 (4.3%)	483 (2.7%)	502 (2.7%)		9 (6.0%)	167 (2.8%)	176 (2.9%)		678 (2.7%)
	6	7 (1.6%)	116 (0.6%)	123 (0.7%)		2 (1.3%)	37 (0.6%)	39 (0.6%)		162 (0.7%)
	7	1 (0.2%)	10 (0.1%)	11 (0.1%)		0 (0.0)	3 (0.1%)	3 (0.0%)		14 (0.1%)
	8	0 (0.0%)	1 (0.0%)	1 (0.0%)		0 (0.0)	0 (0.0)	0 (0.0)		1 (0.0%)
Preoperative mechanical ventilation*	0	307 (69.5%)	16599 (91.7%)	16906 (91.1%)	<0.001	107 (71.8%)	5473 (91.5%)	5580 (91.0%)	<0.001	22486 (91.1%)
	1	135 (30.5%)	1511 (8.3%)	1646 (8.9%)		42 (28.2%)	511 (8.5%)	553 (9.0%)		2199 (8.9%)

Categorical variables are described using frequency (%). Continuous variables are described as the median (range). AAO, Ascending aorta dimension; DAO, Descending aorta dimension; MPA, Main pulmonary artery dimension; MV, Mitral blood flow velocity; TV2, Transtricuspid velocity2; BMI, Body mass index; HCT, Hematocrit; LVDD, Left ventricular end diastolic dimension; LVEF, Left ventricular ejection fraction; LVFS, Left ventricular fraction shortening; LVPWD, Left ventricular posterior wall dimension; AI, Aortic insufficiency; MR, Mitral regurgitation; PI, Pulmonary insufficiency; TR, Tricuspid regurgitation; RDW-SD, Red blood cell volume distribution width-standard deviation; ALP, Alkaline phosphatase; MHC/MCH, Mean hemoglobin content/mean corpuscular hemoglobin; ALT, Alanine aminotransferase; MHC/MCHC, Mean hemoglobin concentration/mean corpuscular hemoglobin concentration; APTT, Activated partial thromboplastin time; TFIC, Tissue factor induced coagulation; TGA, transposition of the great arteries; APVC, anomalous pulmonary venous connection; COA, coarctation of the aorta; IAA, interrupted aortic arch; AVC, atrioventricular canal; DORV, double-outlet right ventricle; ASD, atrial septal defect; PDA, patent ductus arteriosus; PFO, patent foramen ovale; TOF, tetralogy of Fallot; VSD, ventricular septal defect. Note: variables marked with * are included in the XGBoost model.

Comparative analysis of in-hospital mortality and risk categories for each procedure are listed in Table 2. The most common procedures included ventricular septal defect (VSD) membranous repair, tetralogy repair, and VSD subarterial repair. The RACHS-1 categories consist of six groups labeled 1–6, and the STS-EACTS categories consists of five groups labeled 1–5, a higher number means a higher mortality risk. The risk of mortality associated with each procedure was calculated. The in-hospital mortality rate for each procedure ranged from 0–75%, and no death was recorded in 31 procedures. Mortality rates and risk stratification for specific procedures were also estimated using a Bayesian random effects model.

**Table 2.** **Comparative analysis of in-hospital mortality and risk categories for procedures**.

Procedure	Cases (n)	Death (n)	Mortality (unadjusted)	Mortality (model* based)	Risk stratification	RACHS-1 Grade	STS-EACTS Grade
ASD hybrid repair (off pump)	87	0	0%	0.46%	1	2	2
ASD secundum repair	551	1	0.18%	0.13%	1	1	1
ASD secundum repair (patch)	1338	1	0.07%	0.14%	1	1	1
AVSD repair - depression	391	3	0.77%	0.42%	1	3	3
AVSD repair - single patch	116	1	0.86%	0.39%	1	3	3
Aortic valvuloplasty	109	1	0.92%	0.40%	1	2	2
Coronary art. fistula repair	78	0	0%	0.50%	1	2	2
Cortraitriatum repair	70	0	0%	0.50%	1	3	2
Mitral valvuloplasty	461	7	1.52%	0.85%	1	3	2
PAPVD isolated repair	115	0	0%	0.37%	1	1	1
Pulm. Infundibulum resection (indirect)	178	0	0%	0.31%	1	2	1
Subaortic fibromyectomy	155	0	0%	0.32%	1	3	1
Subaortic myectomy	80	1	1.25%	0.52%	1	3	1
Tricuspid valvuloplasty	540	3	0.56%	0.77%	1	3	2
VSD canal type repair	154	0	0%	0.33%	1	3	1
VSD membranous repair	7910	23	0.29%	0.33%	1	2	1
VSD subarterial repair	1641	1	0.06%	0.12%	1	2	1
Vascular ring repair	132	0	0%	0.37%	1	2	1
A-P window repair	31	1	3.23%	0.79%	2	2	2
A-V fistula repair	6	1	16.67%	1.53%	2	2	2
ASD common atrium repair	9	0	0%	1.27%	2	2	2
ASD repair - minimal invasive & CPB	29	0	0%	0.81%	2	2	2
ASD sinus venosus repair	38	0	0%	0.67%	2	1	2
Aortic valvotomy	56	0	0%	0.61%	2	2	2
Asc. aorta patch aortoplasty	184	6	3.26%	1.97%	2	2	2
Coarct repair - bypass or tubular graft	4	0	0%	1.53%	2	1	1
Coarct. repair - resection & E to S	377	10	2.65%	1.64%	2	1/2	2
Coarct. repair - patch aortoplasty	253	4	1.58%	1.92%	2	1/2	3
DORV repair - IVR	375	12	3.20%	3.09%	2	3	4
Ebstein anomaly repair	106	1	0.94%	1.17%	2	3	4
Excision of cardiac tumor	96	2	2.08%	2.39%	2	3	4
Exicion of intracardiac vegetation	11	0	0%	1.22%	2	2	2
Fontan operation - I stage	110	4	3.64%	2.97%	2	3	2
Hemitruncus repair	21	0	0%	1.02%	2	4	2
PDA closure (CPB)	32	0	0%	0.82%	2	1	2
PDA closure (off pump)	386	6	1.55%	1.62%	2	1	2
Pacemaker - re-implant	8	0	0%	1.34%	2	1	1
Pericardectomy	20	0	0%	0.99%	2	1	2
Pericardial drainage	160	8	5%	3.26%	2	1	4
Pulm. Infundibulum incision & resection	46	1	2.17%	1.99%	2	2	1
Pulmonary art. sling repair	20	0	0%	0.96%	2	3	3
Pulmonary art. stent	13	0	0%	1.17%	2	2	2
Pulmonary arterioplasty	251	8	3.19%	3.42%	2	2	2
Pulmonary valvotomy	200	3	1.50%	1.29%	2	2	2
Pulmonary valvotomy - hybrid	5	0	0%	1.43%	2	2	2
Subaortic septal patch (Konno)	15	0	0%	1.05%	2	4	3
Supravalve mitral ring resection	28	0	0%	0.79%	2	3	2
Systemic vein repair	14	0	0%	1.12%	2	2	3
TAPVD repair - intracardiac	184	2	1.09%	1.32%	2	2/4	4
TAPVD repair - mixed type	34	1	2.94%	2.67%	2	2/4	4
Tetralogy repair	2392	46	1.92%	1.95%	2	2	2
Tricuspid replacement (mech.)	13	0	0%	1.17%	2	3	2
VSD Hybrid repair (off pump)	171	1	0.58%	0.84%	2	2	2
VSD hybrid repair (CPB)	21	0	0%	0.99%	2	2	2
VSD multiple repair	127	2	1.57%	1.75%	2	2	2
VSD muscular repair	105	1	0.95%	1.20%	2	2	1
VSD repair - minimal invasive & CPB	45	0	0%	0.71%	2	2	2
AVSD repair - two patches	269	15	5.58%	5.27%	3	3	3
Ao translocation operation	17	2	11.76%	4.60%	3	3	3
Aortic arch repair	70	5	7.14%	6.30%	3	4	4
Aortic valve replacement (mech.)	45	3	6.67%	4.46%	3	3	1
Cavopulmonary shunt - bilateral	102	6	5.88%	4.61%	3	2	2
DOLV repair	4	0	0%	2.05%	3	3	4
Delayed sternal closure	891	51	5.72%	6.23%	3	1	1
Double switch (Senning + Rastelli)	3	0	0%	1.80%	3	4	5
Excision of cardiac diverticulum	4	0	0%	1.53%	3	2	2
Fontan operation - II stage	537	30	5.59%	5.77%	3	3	2
Kawashima procedure	4	0	0%	2.05%	3	3	1
Mitral replacement (mech.)	96	11	11.46%	7.86%	3	3	4
PA banding	101	12	11.88%	3.78%	3	3	4
Pulm. infundibulum resection & patch	78	4	5.13%	4.48%	3	2	1
Pulm. infundibulum resection, patch across annulus	292	15	5.14%	6.09%	3	2	2
Pulm. vein stenosis repair	40	2	5%	5%	3	4	4
Pulmonary atresia/IVS repair	19	2	10.53%	4.87%	3	4	3
Pulmonary atresia/VSD repair	288	17	5.90%	6.24%	3	4	3
Pulmonary valvotomy (off pump)	18	1	5.56%	4.60%	3	2	2
Senning procedure	19	1	5.26%	4.34%	3	3	4
TAPVD repair - supracardiac	238	13	5.46%	5.77%	3	2/4	4
ALC-PA repair	58	10	17.24%	14.97%	4	3	2
Cavopulmonary shunt - left	83	11	13.25%	12.68%	4	2	1
Cavopulmonary shunt - right	359	35	9.75%	10.85%	4	2	1
Central shunt - with graft	38	8	21.05%	11.02%	4	3	4
Conduit RV - PA	72	13	18.06%	13.98%	4	2	3
Double Switch (Hemi-Mustard)	6	1	16.67%	9.32%	4	4	5
Interrupted aortic arch repair	77	11	14.29%	13.32%	4	5	4
Interruption of bronchial collaterals	8	1	12.50%	9.32%	4	1	2
Pacemaker - primary implant	17	2	11.76%	11.95%	4	1	1
R.E.V. RV - PA connection	7	1	14.29%	8.28%	4	4	3
Rastelli operation	50	6	12%	9.44%	4	4	3
Rt. or lt. heart assist	8	3	37.50%	9.32%	4	2	1
TAPVD repair - infracardiac	43	6	13.95%	14.74%	4	2/4	4
Takedown previous shunt	11	1	9.09%	6.80%	4	2	3
Arterial switch repair	456	87	19.08%	19.20%	5	3	3
Atrial septectomy	12	4	33.33%	27.36%	5	4	4
Coronary artery repair	6	2	33.33%	24%	5	3	2
DKS connection	9	4	44.44%	30.71%	5	6	5
Double switch (Senning + ASO)	18	6	33.33%	18.03%	5	4	5
Fontan takedown	9	5	55.56%	41.71%	5	3	3
Norwood operation	4	3	75%	50.76%	5	6	5
PA debanding	3	1	33.33%	16.53%	5	3	4
PA unifocalization	61	11	18.03%	21%	5	4	4
Truncus repair	33	7	21.21%	20.44%	5	4	4

ASD, Atrial septal defect; AVSD, Atrioventricular septal defect; PAPVD, Partial anomalous pulmonary venous drainage; VSD, Ventricular septal defect. A-P window, Aorto-pulmonary window; A-V fistula, Arteriovenous fistula; DORV, Double outlet right ventricle; CPB, Cardiopulmonary bypass. PDA, Patent ductus arteriosus; TAPVD, Total anomalous pulmonary venous drainage; ALC-PA, Anomalous left coronary artery from pulmonary artery. RV, Right ventricle; PA, Pulmonary artery; DKS connection, Damus–Kaye–Stansel (DKS) procedure; ASO, Arterial switch operation. *: Bayesian random effect model.

### 3.2 Prediction Value of the Different Models

**Fig. 2.**
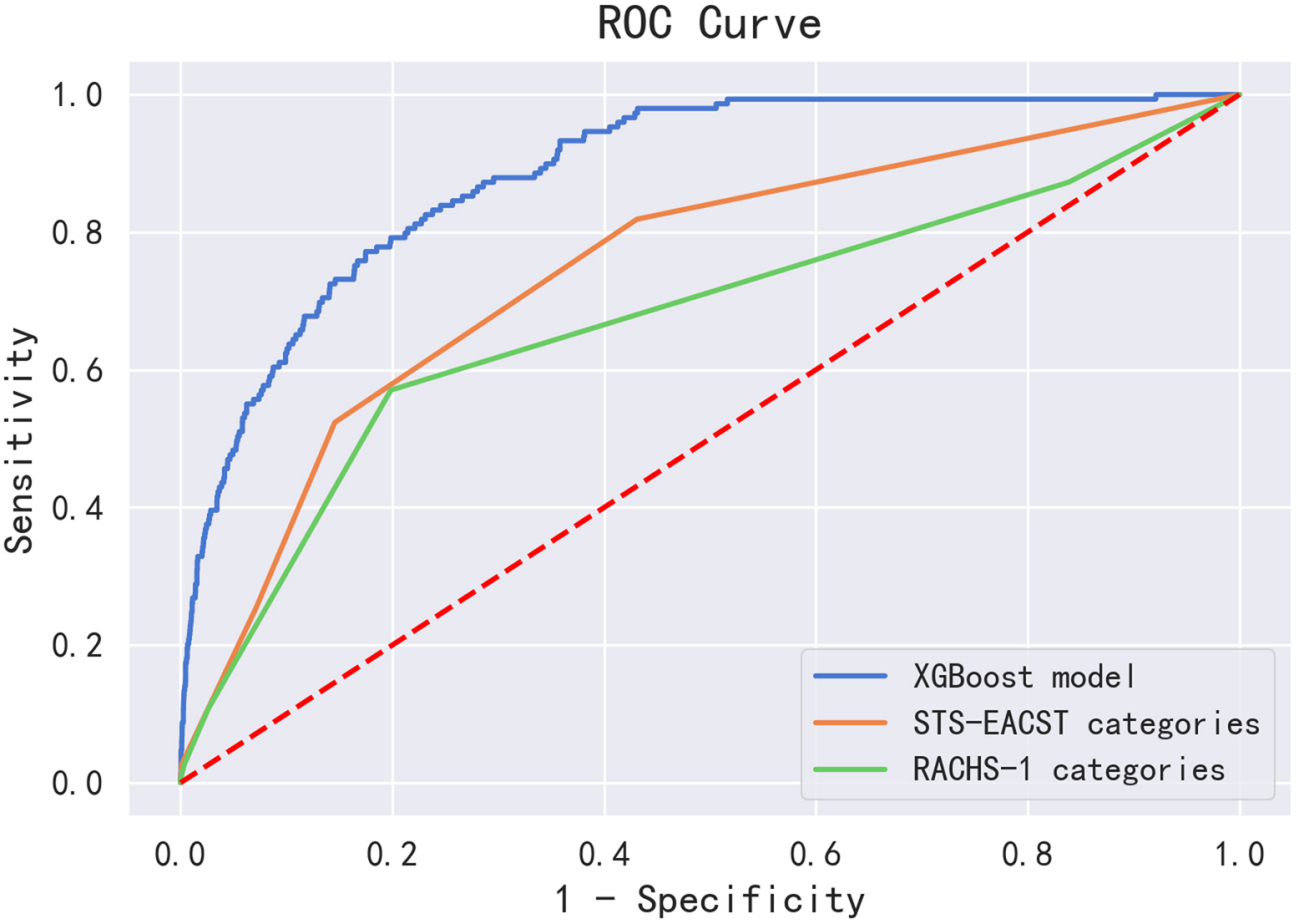
**Comparison of the prediction values of the XGBoost model, STS-EACTS categories, and RACHS-1 categories in the testing set**.

The performance of the XGBoost model, STS- EACTS, and RACHS-1 risk stratification categories in the testing set are shown in Table 3 and Fig. 2. The AUC of the STS-EACTS and RACHS-1 risk stratification categories was 0.748 (95% CI: 0.707–0.789) and 0.677 (95% CI: 0.627–0.728), respectively. Our XGBoost model yielded the best AUC (AUC = 0.887, 95% CI: 0.866–0.907), with a sensitivity and specificity of 0.785 and 0.824, respectively (Table 3).

**Table 3.** **Prediction value of the model based on the XGBoost model, STS-EACTS categories, and RACHS-1 categories in the testing set**.

Model	AUC	AUC 95% CI	*p*-value	Sensitivity	Specificity
XGBoost model	0.874	(0.848, 0.901)	<0.001	0.751	0.879
STS-EACTS categories	0.748	(0.707, 0.789)	<0.001	0.819	0.569
RACHS-1 categories	0.677	(0.627, 0.728)	<0.001	0.570	0.801

### 3.3 Importance of the Top 10 Variables in the Prediction of the XGBoost Model

The top 10 variables that contribute the most to the prediction power of the model are listed in Table 4. The higher the weight coefficient of a feature, the more significant role it plays in the model for outcome classification. The weight coefficient for saturation of pulse oxygen categories, risk categories, age, preoperative mechanical ventilation, atrial shunt, pulmonary insufficiency, ventricular shunt, left atrial dimension, a history of cardiac surgery, numbers of defect were 0.10638574, 0.07759346, 0.07303152, 0.07014898, 0.065226465, 0.05785214, 0.05760804, 0.052233107, 0.051096234, 0.0437589, respectively (Table 4). The excluded variables are listed in **Supplementary Table 1**.

**Table 4.** **Importance of the top 10 risk factors in the prediction of the XGBoost model**.

Feature	Weight coefficient
Saturation of pulse oxygen categories	0.106386
Risk categories	0.077593
Age	0.073032
Preoperative mechanical ventilation	0.070149
Atrial shunt	0.065226
Pulmonary insufficiency	0.057852
Ventricular shunt	0.057608
Left atrial dimension	0.052233
A history of cardiac surgery	0.051096
Number of defects	0.043759

Weight coefficient: Refer to the extent to which each indicator contributes to the model.

## 4. Discussion

Prediction of in-hospital mortality risk is clinically important for directing patient postoperative management. In our study, we compared the XGBoost model with traditional tools for predicting mortality in pediatric CHD surgery. To our knowledge, this is the first study to compare machine learning algorithms with the RACHS-1 and STS-EACTS categories for the prediction of in-hospital mortality risk in pediatric CHD surgery. And we found that in children with CHD of China, the XGBoost model was more accurate in predicting in-hospital mortality for CHD surgery than in the RACHS-1 and STS-EACTS categories.

In our study, the in-hospital mortality rate after CHD surgery was 2.4%, which is consistent with that previously reported in China [5] and in western countries [25, 26, 27], but much lower than that reported in developing countries [28, 29]. Cardiac surgeons use traditional tools such as the RACHS-1 and STS-EACTS to report patient outcomes. The RACHS-1 categories were constructed based on a combination of the opinions of 11 experts and empirical data to predict in-hospital mortality [12, 30]. The RACHS-1 categories classifies procedures into six levels of risk of mortality based on a few clearly defined criteria. In previous studies, the risk of mortality from CHD surgery with different RACHS-1 stratification ranged from 0.26% to 62% [31, 32, 33], which is consistent with the findings of our study. An objective, empirically based tool named STS-EACTS, without the input of an expert panel, has been developed for analyzing in-hospital mortality associated with CHD surgery [14, 34]. However, the RACHS-1 and STS-EACTS categories only consider procedural characteristics and ignore individual patient characteristics. Both categories lack precision when estimating the risk for individual patients. In addition, some procedures (19%) in our cohort could not be classified using the RACHS-1 or STS-EACTS categories. Due to the enormous differences in patient-related variables and CHD surgery, it is difficult to establish a mortality risk prediction model.

Hence, one of the greatest challenges in medicine is how to deal with individuals who have the same disease but with different manifestations. Machine learning algorithms may provide a solution for this problem. Furthermore, previous study further demonstrated that machine-learning methods, especially gradient boosting models, are promising to outperform existing clinical risk scores [35]. Machine learning models have currently been explored as a tool to predict mortality, morbidity, and complications in patients with CHD [22, 36, 37]. A XGBoost model was constructed based on surgical risk stratification and patient preoperative variables in this study. According to previous studies, the AUC of the STS-EACTS and RACHS-1 categories to predict the mortality and complications of CHD surgery in children was 0.68–0.79 [14, 22, 28, 38, 39]. In our study, the AUC of the XGBoost model was 0.887, which was better than that of the STS-EACTS (AUC = 0.748) and RACHS-1 (AUC = 0.677) categories. The results showed that the XGBoost model was able to predict in-hospital mortality with improved predictive power compared to the STS-EACTS and RACHS-1 models.

In addition to surgical risk stratification, patient-related variables also had a significant impact on the performance of the postoperative mortality risk prediction model. The XGBoost model incorporates demographic characteristics, preoperative echocardiography characteristics, and laboratory examination results into the final predictive model. We analyzed the importance of these risk factors in the XGBoost model. Our results showed that saturation of pulse oxygen categories had the greatest impact on the predictive performance of the model. Previous studies have shown that oxygen saturation correlates with mortality in children undergoing CHD surgery [40, 41]. A decrease in oxygen saturation at 24 hours after the operation may increase the mortality rate of newborns with cyanotic cardiopathies [42].

For patients with CHD who have received cardiac intervention, preoperative mechanical ventilation and RACHS categories may affect their mortality during hospitalization [43]. Mechanical ventilation has been shown to be a strong predictor of in-hospital mortality in children with noncardiac surgery [44]. In addition, study has found that newborns with severe CHD may have increased mortality if they need unplanned cardiac reintervention, and according to the results of multifactorial analysis, mechanical ventilation before heart intervention and the larger RACHS-1 category are independent risk factors for unplanned cardiac re-intervention [45]. In this study, we also found that risk categories and preoperative mechanical ventilation are top influencing factors of the predictive performance in XGBoost model. This reminds clinicians to pay more attention to the prognosis of these patients whose surgery is at greater risk category or who require preoperative mechanical ventilation.

Age, as an important factor in the mortality rate of children with CHD, has also been mentioned in several studies. A study in Taiwan Province of China found that the majority (i.e., more than 90%) of CHD deaths occur within the first 5 years of life (mainly in infancy) [46]. The study showed that the mortality rate of CHD patients has a downward trend with the increase of age [47]. Our study also found that age is an influencing factor of in-hospital mortality in children with CHD and made a great contribution to the model. Clinicians should pay more attention to younger CHD patients in practice.

In addition to the above factors, the results of the machine learning model in this study also show that atrium shunt and ventricular shunt may also be the factor of in-hospital mortality. The reason may be that these factors affect the occurrence of postoperative complications in patients. Low cardiac output syndrome (LCOS) is a common life-threatening postoperative complication of heart disease that may contributes to postoperative morbidity and mortality [48, 49, 50, 51]. Atrial shunt and ventricular level shunt were all independent risk predictors of LCOS [52]. Bangrong Song *et al*. [51] believed that more attention should be paid to CHD patients age ≤4 years, preoperative oxygen saturation ≤93%, CPB duration ≥60 minutes, two-way ventricular shunt, postoperative residual shunt to improve the prognosis of these patients. In addition, study has found that left atrium dimension is associated with long-term adverse outcomes (hospitalization due to heart failure, all-cause mortality, new-onset atrial fibrillation, and/or embolic stroke during follow-up) of rheumatic heart disease [53].

In this study, we found that the number of defects influences in-hospital mortality in patients with CHD. The reason may be that if the patient carries multiple CHD at the same time, the prognosis is much worse than that of the patient with a single type of CHD. Presently, the most common types of CHDs include ventricular septal defect (VSD), patent ductus arteriosus (PDA), secundum atrial septal defect (ASDII), pulmonary stenosis (PS) and tetralogy of Fallot (TOF), and the incidence of each CHD is different [54]. Several studies have further divided the CHD population into simple and severe CHD groups [54, 55], and it is found that individuals with simple CHD (e.g., VSD or ASD) have higher survival rates, almost similar to normal populations [47]. However, the prognoses of patients with severe CHD varies widely [47].

Clinicians should focus on the top variables in the model to improve patient outcomes by dealing with variables that can be managed. For example, on the premise of ensuring the treatment effect, surgery with low-risk categories should be selected to improve the postoperative survival rate of patients. For the CHD patients with abnormalities in the above top indicators should be paid closer attention to their postoperative recovery status, which is the important significance of this study to propose this model.

This study has some limitations. First, this study is a single-center retrospective study. However, this is the first study to use a machine learning algorithm to predict mortality in pediatric CHD surgery in a large sample. In addition, our center is the most famous treatment center for children with congenital heart disease in China, with patients from most provinces in China. Second, the in-hospital mortality rates recorded in this study may not include all operation-related deaths, and need to include data from patients after discharge. This needs to be addressed in a more complete data source. Third, there was an imbalance in the number of patients who died and survivors in this study, and we did not perform a 1:N case-control match at the time of patient enrollment. During model building, we try to deal with the imbalance of samples in the training set, we tried to process the unbalanced classes and tuned the parameter ‘scale_pos_weight’, a parameter adjusting the balance of positive and negative weights in the XGBoost package. However, this did not improve the predictive performance of the model.

## 5. Conclusions

In conclusion, our single-center study of 24,685 patients demonstrated that using a combination of procedure complexity categories and preoperative patient-level factors, the XGBoost model had higher accuracy in in-hospital mortality prediction than both the RACHS-1 and STS–EACTS categories. In clinical practice, machine learning models can be established based on the surgical database for risk prediction to improve cardiac surgical care.

## Supplementary Material

Supplementary material associated with this article can be found, in the online version, at https://doi.org/10.31083/j.rcm2311376.
