# Supplementary material for: Machine Learning Model for Predicting Risk of In-Hospital Mortality after Surgery in Congenital Heart Disease Patients
Source: Rev Cardiovasc Med. 2022 Nov 3;23(11):376. doi: 10.31083/j.rcm2311376 (PMC11269077; doi:10.31083/j.rcm2311376)
Supplement: Supplementary file 1 [file 2153-8174-23-11-376-s1.docx]

Supplementary Table 1. Excluded variables.

| Total hemoglobin |
| --- |
| Plasma fibrinogen |
| Thrombin time |
| D-Dimer |
| Lactic acid |
| Prealbumin |
| Serum sodium |
| serum potassium |
| Serum chloride |
| Serum calcium |
| Serum abio-phosphorus |
| Serum magnesium |
| Creatine Kinase |
| Partial pressure of oxygen |
| Saturation of blood oxygen |
| Oxyhemoglobin/hemoglobin |
| Partial Pressure of Carbon Dioxide |
| Total Carbon Dioxide Content |
| Troponin-I |
| N-terminal Pro-brain Natriuretic Peptide |
| C-reaction protein |
| IgG |
| IgA |
| IgM |
| HBSAg |
| HBeAg |
| Anti-HCV IgG |
| Treponema pallidum antibody |
| Serum procalcitonin |
| Cytomegalovirus-IgG |
| Cytomegalovirus-IgM |
| Mycoplasma Pneumoniae Antibodies |
| Human chorionic gonadotrophin-β |
| Serum bicarbonate |
| Blood glucose |
| Blood urea nitrogen |
| Anti-HBc |
| Anti-HBe |
| Anti-HBs |
| Carbondioxide combining power |
| Left ventricular end-systolic dimension |
| Left ventricular outflow tract |
| Left ventricular posterior walldepth |
| Left ventricular posterior wall thickness at end-systole |
| Right ventricular outflow tract |
